# Outcome prediction in chronic unilateral lumbar radiculopathy: prospective cohort study

**DOI:** 10.1186/s12891-015-0474-9

**Published:** 2015-02-07

**Authors:** Trond Iversen, Tore K Solberg, Tom Wilsgaard, Knut Waterloo, Jens Ivar Brox, Tor Ingebrigtsen

**Affiliations:** Bindal Legekontor, Terråk, Norway; Department of Physical Medicine and Rehabilitation, University Hospital of North Norway, Tromsø, Norway; Department of Ophthalmology and Neurosurgery, University Hospital of North Norway, Tromsø, Norway; The Norwegian Registry for Spine Surgery (NORspine), North Norway Regional Health Authority, Tromsø, Norway; Department of Community Medicine, Faculty of Health Sciences, UiT The Artic University of Norway, Tromsø, Norway; Department of Neurology, University Hospital of North Norway, Tromsø, Norway; Department of Psychology, Faculty of Health Sciences, UiT The Artic University of Norway, Tromsø, Norway; Department of Physical Medicine and Rehabilitation, Oslo University Hospital, University of Oslo, Oslo, Norway; Department of Clinical Medicine, Faculty of Health Sciences, UiT The Artic University of Norway, Tromsø, Norway

**Keywords:** Chronic unilateral lumbar radiculopathy, Lumbar nerve root impingement, Outcome prediction, Radiculopathy, Sciatica

## Abstract

**Background:**

Identification of prognostic factors for persistent pain and disability are important for better understanding of the clinical course of chronic unilateral lumbar radiculopathy and to assist clinical decision-making. There is a lack of scientific evidence concerning prognostic factors. The aim of this study was to identify clinically relevant predictors for outcome at 52 weeks.

**Methods:**

116 patients were included in a sham controlled clinical trial on epidural injection of glucocorticoids in patients with chronic unilateral lumbar radiculopathy. Success at follow-up was ≤17.5 for visual analogue scale (VAS) leg pain, ≤22.5 for VAS back pain and ≤20 for Oswestry Disability Index (ODI). Fifteen clinically relevant variables included demographic, psychosocial, clinical and radiological data and were analysed using a logistic multivariable regression analysis.

**Results:**

At follow-up, 75 (64.7%) patients had reached a successful outcome with an ODI score ≤20, 54 (46.6%) with a VAS leg pain score ≤17.5, and 47 (40.5%) with a VAS back pain score ≤22.5.

Lower age (OR 0.94 (CI 0.89–0.99) for each year decrease in age) and FABQ Work ≥34 (OR 0.16 (CI 0.04-0.61)) were independent variables predicting a successful outcome on the ODI.

Higher education (OR 5.77 (CI 1.46–22.87)) and working full-time (OR 2.70 (CI 1.02–7.18)) were statistically significant (P <0.05) independent predictors for successful outcome (VAS score ≤17.5) on the measure of leg pain. Lower age predicted success on ODI (OR 0.94 (95% CI 0.89 to 0.99) for each year) and less back pain (OR 0.94 (0.90 to 0.99)), while higher education (OR 5.77 (1.46 to 22.87)), working full-time (OR 2.70 (1.02 to 7.18)) and muscle weakness at baseline (OR 4.11 (1.24 to 13.61) predicted less leg pain, and reflex impairment at baseline predicted the contrary (OR 0.39 (0.15 to 0.97)).

**Conclusions:**

Lower age, higher education, working full-time and low fear avoidance beliefs each predict a better outcome of chronic unilateral lumbar radiculopathy. Specifically, lower age and low fear avoidance predict a better functional outcome and less back pain, while higher education and working full-time predict less leg pain. These results should be validated in further studies before being used to inform patients.

**Trial registration:**

Current Controlled Trials ISRCTN12574253. Registered 18 May 2005.

## Background

Radiculopathy, or sciatica, is defined as radiating leg pain below knee level with neurological deficits in the distribution of the lumbosacral nerves [1,2]. The most common cause of radiculopathy is lumbar disc herniation [3,4]. Annual prevalence rates vary widely from 2 to 34%, probably due to differences in the definition of symptoms and interpretation of clinical findings [2,5,6].

The natural course of radiculopathy also varies between studies, as do the success rates after treatment, both depending on the inclusion criteria and outcome measures used [7]. For example, a study on primary care patients indicated a good prognosis, with approximately 75% of the patients experiencing full recovery after 3 months [8]. In a study of patients who were referred to hospital, nearly 70% had persistent symptoms 13 years later [9].

Previous studies have assessed many possible predictors associated with the prognosis of radiculopathy, such as clinical, demographic, psychosocial and work-related risk factors, radiological findings and treatment modalities [10,11]. Female gender [12], symptoms of depression and anxiety [13], psychosomatic symptoms [14], long-lasting leg pain, carrying heavy loads, driving at least 2 hours per day [15], and positive nerve stretch tests are among the numerous factors reported to be associated with a less favourable outcome [8,16].

Two recent systematic reviews attempted to synthesize the evidence on prognostic factors for sciatica [17,18]. Heterogeneity of the included studies precluded pooling of results and meta-analysis in both reviews. The review by Ashworth et al. [17] included eight studies of non-surgically treated patients. No strong or consistent predictor for persistent disability could be identified, but clinical, occupational and individual factors were found to be more strongly associated with outcome than psychological factors in sciatica populations. The authors recommended that prospective studies with high methodological quality (multivariable models) using a well-defined and consistent definition of radiculopathy should be performed, and that psychosocial, clinical and radiological data should be included in risk factor analyses. The review by Verwoerd et al. [18] screened 168 articles and included 23 studies. Only nine articles reported results from multivariable analysis [8,12,19-25]. Most articles reported results from studies of patients in secondary care, and the diagnosis of sciatica was frequently based on clinical criteria only. The review included surgery as outcome and found that only high leg pain intensity at baseline was strongly associated with subsequent surgery. The authors commented that clinical decision-making is hampered by lack of scientific evidence concerning prognostic factors.

To study possible predictors for outcome, validated patient-reported outcome measures should be used with standardized cut-offs that distinguish between success and non-success [26]. In this study, we used validated cut-offs on the Oswestry Disability Index (ODI) and visual analogue scales (VAS) for leg and back pain [27-30].

In summary, the reviews on predictors referred to above for the study of outcome of sciatica have identified a limited number of variables of clinical importance but the studies vary in the use of inclusion criteria and outcome measures, use unclear definitions of success criteria, and use statistical methods inconsistently. In the present study of chronic unilateral lumbar radiculopathy, we included a homogeneous patient sample selected with clear inclusion criteria in a specialized care setting, and clinically relevant outcome measures with well-defined cut-offs for successful outcomes. The aim of this study was to identify clinically relevant predictors for outcome among patients with chronic radiculopathy.

## Methods

### Setting

The study was performed as part of a multicentre randomized controlled trial (RCT) on the treatment effect of caudal epidural injections for chronic unilateral lumbar radiculopathy [31], and as part of a study on the association between findings at clinical examination and lumbar nerve root impingement [32]. We used the Oswestry Disability Index (ODI) and the Visual Analogue Scale (VAS) score for low back pain and leg pain as outcome measures in the RCT. The treatment intervention in the RCT had no short or long-term effect on chronic unilateral lumbar radiculopathy. This allowed the use of the trial data in this study [33].

### Patients

Eligible patients with suspected chronic unilateral lumbar radiculopathy, aged between 20 and 60 years, referred to outpatient multidisciplinary back clinics of five Norwegian hospitals, were consecutively assessed for inclusion. The inclusion period was 3 years, between 2005 and 2009. 461 patients with suspected chronic unilateral lumbar radiculopathy were assessed for inclusion: 376 (81.6%) were referred from general practitioners and 85 (18.4%) were internally referred in the participating hospitals.

The inclusion criterion was chronic unilateral lumbar radiculopathy lasting more than 12 weeks. The intensity of the leg pain, radiating from the back to below the knee, had to be comparable to or worse than the back pain. A clinical examination was carried out by trained physicians and physiotherapists. The assessment included muscle strength, sensory loss, reflexes of the Achilles tendon and patella, and the straight leg raising test. The results of each clinical test were dichotomized as normal or abnormal as described previously [32]. These inclusion criteria ensured a homogeneous patient population with clinically verified chronic unilateral lumbar radiculopathy. Magnetic resonance imaging (MRI) in 109 (94.0%) or computer tomography (CT) in 7 (6.0%) patients was used to specifically clarify whether the nerve root in question was impinged or not. Two experienced neuroradiologists evaluated all MRI and CT scans. They were not provided any clinical information and had not been involved in the selection or care of the included patients. There were no requests for a correspondence between demonstrated level of radiculopathy by clinical examination and findings on imaging.

We excluded 345 (74.8%) patients fulfilling predefined exclusion criteria according to the original RCT: 146 (42.3%) due to unspecific low back pain with referred leg pain, 105 (30.4%) due to radiculopathy improving during the last 2 weeks before the inclusion examination, 24 (7.0%) due to radiculopathy requiring necessary urgent referral to surgery, 16 (4.6%) because of back surgery prior to this study, 37 (10.7%) due to different medical conditions (pregnancy, breastfeeding, use of anticlotting medication), and 17 (4.9%) because they declined to participate.

At this point, 116 patients with chronic unilateral lumbar radiculopathy were included in the study. At all study sites the patients received standardized oral and written information about spine anatomy and function at baseline and follow-up. They were encouraged to engage in physical activity, and all patients received the brochure ‘Worth knowing about bad backs. What experts agree on’ [34]. The decision about surgery during follow-up was made for individual patients at each centre, and no standardized criteria were established for surgical treatment. 99 (85.3%) of the included patients were followed up at 52 weeks. Written informed consent was obtained and the Regional Committee for Medical and Health Research Ethics in North Norway approved the study.

### Procedure and measurements

At baseline, a questionnaire on sociodemographic factors, fear avoidance belief (FABQ), duration of low back pain and leg pain and outcome measures was completed by the patients.

#### Outcome measures

We used functional status assessed with the ODI as the primary outcome measure and leg pain and back pain as secondary outcome measures. At follow-up after 52 weeks the ODI score, the VAS leg pain and the VAS back pain were registered. A successful outcome score was set to ≤17.5 for VAS leg pain, ≤22.5 for VAS back pain and ≤20 for ODI, as recommended by Haugen et al. after Receiver Operating Curves (ROC) analysis of outcomes in 466 patients [30]. Change scores were calculated as difference between baseline and follow-up scores [35,36].

The ODI contains 10 questions on limitations of daily living activities [37-39]. Each variable was rated on a 0 to 5-point scale, added up, and converted into a percentage score. The range of possible values is from 0 to 100 (where 0 = no disability). Leg pain and low back pain were measured using the VAS 0–100 (where 0 = no pain).

#### Predictors for outcome

Table 1 shows that we analysed sociodemographic variables, psychological variables, pain history, findings from clinical examination, and imaging as possible predictors. These were predefined based on findings in previous literature, including results reported from the Norwegian Registry for Spine Surgery [40,41] and our assessment of clinical relevance. Age, duration of leg and back pain and body mass index were analysed as continuous variables. Gender, current smoking, university or college education, working full-time, positive straight leg test, presence of muscle weakness, sensory loss or reflex impairment, concordance between nerve root impingement on MRI and clinical radiculopathy, presence of Modic type I or II changes and FABQ [42] were dichotomized. We chose ≥34 as cut-off for an elevated fear avoidance belief for the FABQ subscale for work (FABQW) [43] and ≥15 for the FABQ subscale for physical activity (FABQPA) [44].

**Table 1:** **Characteristics of the patients (n = 116) at baseline**

*Sociodemographic variables*	
Age years, mean (SD)	42.0 (10.3)
Male gender, n (%)	68.0 (58.6)
Current smoker, n (%)	49.0 (42.2)
University or college education, n (%)	22.0 (19.0)
Working full-time, n (%)	43.0 (37.1)
*Low back pain/sciatica history/fear avoidance*	
Low back pain weeks, mean (SD)	53.4 (110.0)
Leg pain weeks, mean (SD)	42.0 (99.0)
Fear avoidance belief questionnaire about work, mean (SD)	12.8 (5.0)
Fear avoidance belief questionnaire about physical activity, mean (SD)	23.4 (10.2)
*Clinical examination*	
Straight leg raising <60°, n (%)	62.0 (53.4)
Muscle weakness, n (%)	94.0 (81.0)
Dermatomal sensory loss, n (%)	83.0 (71.6)
Reflex impairment, n (%)	55.0 (47.4)
Body mass index, mean (SD)	26.3 (3.8)
*Magnetic resonance or CT imaging*	
Concordance between nerve root impingement on MRI and clinical radiculopathy n (%)	60 (51.7)
Modic type I and I/II, n (%)	66.0 (56.9)

### Statistical analysis

We calculated means and standard deviations (SD) for continuous variables, and frequencies and proportions for categorical variables. Paired samples t-tests were used to test change scores between baseline and follow-up for patient-reported outcomes. ANalysis Of VAriance (ANOVA) was used to compare mean differences between groups. We used univariable and stepwise backward (Wald) multivariable binary logistic regression to analyse associations between predictors and outcome measures. Predictors with P value <0.20 from the univariable analysis were used in the multivariable analysis. In the analysis we adjusted for the baseline values. Odds ratios (ORs) with 95% confidence intervals (CI) were calculated. P values <0.05 were considered statistically significant. All analyses were performed using the Statistical Package for the Social Sciences (SPSS) software version 22 (IBM Software, NY, USA).

## Results

In total, 116 patients with chronic unilateral lumbar radiculopathy were included. Their clinical and demographic characteristics are summarized in Table 1. All 15 variables were included in the subsequent predictor analysis. We defined high correlation between prognostic factors to be >0.60. Duration of leg pain and back pain were highly correlated (Spearman’s ρ = 0.71) and duration of back pain was therefore not included in the analysis.

Table 2 shows that there was a statistically significant (P < 0.001) mean improvement for both the ODI and the VAS leg pain and VAS back pain outcome measures from baseline to follow-up after 52 weeks. The mean improvement was substantial (VAS decrease ≥20) for leg pain.

At follow-up, 75 (64.7%) of the patients had reached a successful outcome with an ODI score ≤20, 54 (46.6%) with a VAS leg pain score ≤17.5, and 47 (40.5%) with a VAS back pain score ≤22.5. These outcome values were used in the multivariable logistic regression analysis.

**Table 2:** **Paired samples t-test for patient-reported measures at baseline and follow-up**

**Patient-reported measures**	**n**	**Baseline**	**Follow-up**	**Change**	**t**	**P**
ODI (0–100)	99	30.0 (13.2)	15.5 (13.3)	14.4 (16.3)	8.84	0.001
Leg pain intensity (VAS 0–100)	97	50.6 (24.7)	23.0 (25.8)	27.5 (31.3)	8.67	0.001
Low back pain intensity (VAS 0–100)	93	47.6 (24.3)	27.9 (24.3)	17.9 (30.6)	5.66	0.001

Numbers are mean (SD).

VAS: 0 = no pain.

ODI: 0 = normal function.

Table 3 shows that lower age (OR 0.94 (CI 0.89–0.99) for each year decrease in age) and FABQ Work ≥34 (OR 0.16 (CI 0.04-0.61)) were independent variables predicting a successful outcome on the ODI in multivariable analysis.

**Table 3:** **Multivariable logistic regression analysis**

**Predictors**	**Successful outcome ODI**		**Successful outcome VAS leg pain**		**Successful outcome VAS back pain**	
	**Absolute value at follow-up ≤20, adjusted for its baseline value**		**Absolute value at follow-up ≤17.5, adjusted for its baseline value**		**Absolute value at follow-up ≤22.5, adjusted for its baseline value**	
	**Univariable**	**Multivariable**	**Univariable**	**Multivariable**	**Univariable**	**Multivariable**
Age (year)	0.95 (0.90–1.00)*	0.94 (0.88–0.99)*	0.98 (0.94–1.02)		0.95 (0.91–0.99)*	0.93 (0.89–0.98)*
Male gender	0.63 (0.23–1.77)		0.84 (0.37–1.95)		0.82 (0.35–1.91)	
Current smoker	1.26 (0.44–3.55)		1.06 (0.45–2.48)		1.20 (0.51–2.81)	
University or college education	5.90 (0.72–48.60)**		4.24 (1.23–14.63)*	5.77 (1.46–22.87)*	2.64 (0.84–8.26)**	
Working full-time	2.10 (0.67–6.56)		2.61 (1.07–6.34)*	2.70 (1.02–7.18)*	1.75 (0.74–4.12)**	2.77 (1.02–7.56)*
Leg pain duration (4wk)	0.97 (0.93–1.01)**		0.95 (0.89–1.01)**		0.98 (0.93–1.03)	
Straight leg raising <60°	1.03 (0.38–2.77)		0.97 (0.43–2.19)		1.41 (0.61–3.23)	
Muscle weakness (yes)	0.72 (0.20–2.41)		3.32 (1.12–9.81)*	4.11 (1.24–13.61)*	1.70 (0.58–4.97)	
Dermatomal sensory loss (yes)	1.22 (0.41–3.69)		1.02 (0.42–2.48)		0.79 (0.32–1.96)	
Reflex impairment (yes)	0.50 (0.18–1.40)**		0.40 (0.17–0.92)*	0.39 (0.15–0.97)*	0.90 (0.93–2.07)	
Body mass index (kg/m^2^)	0.92 (0.81–1.04)**		1.04 (0.93–1.17)		1.04 (0.92–1.17)	
Concordance between nerve root impingement on MRI and clinical radiculopathy	0.63 (0.23–1.77)		1.04 (0.46–2.37)		0.93 (0.40–2.16)	
Modic type I and I/II (yes)	0.35 (0.12–1.05)**		0.68 (0.30–1.56)		0.72 (0.31–1.66)	
FABQW ≥34 at baseline	0.27 (0.09–0.85)*	0.16 (0.04–0.61)*	0.38 (0.13–1.07)**		0.34 (0.11–1.07)**	
FABQPA ≥15 at baseline	0.38 (0.14–1.07)**		0.44 (0.19–1.03)**		0.33 (0.14–0.81)*	0.31 (0.11–0.85)*

Odds ratio for successful outcome on ODI and VAS leg and back pain. 95% confidence interval in brackets.

*P < 0.05; **P < 0.20; wk = week.

Table 3 also shows predictors for the secondary outcome measures VAS leg pain and VAS back pain. Higher education (university or college level) (OR 5.77 (CI 1.46–22.87)) and working full-time (OR 2.70 (CI 1.02–7.18)) were statistically significant (P < 0.05) independent predictors for a successful outcome (VAS score ≤17.5) on the measure of leg pain. The presence of muscle weakness (OR 4.11 (CI 1.24–13.61)) also predicted a VAS score for leg pain ≤17.5, while the presence of reflex impairment predicted the contrary (OR 0.39 (CI 0.15–0.97)).

Lower age (OR 0.94 (CI 0.90–0.99) for each year decrease in age) and working full-time (OR 2.77 (CI 1.02-7.56)) predicted a successful outcome (VAS score ≤22.5) for back pain, while FABQ Physical activity ≥15 (OR 0.31 (CI0.11-0.85)) predicted the contrary.

Fifteen (13%) patients underwent surgical decompression of the clinically affected nerve root during follow-up, and outcome data for 12 of them were available. A subanalysis comparing operated and non-operated patients showed that the operated patients had significantly higher baseline scores for ODI, VAS leg pain and VAS back pain and improved significantly more. There were, however, no differences between the groups with regard to the ODI and the VAS leg pain and VAS back pain scores at 52 weeks follow-up (Table 4).

**Table 4:** **ANOVA – difference in outcome scores between patients who did and did not undergo surgical decompression of lumbar spinal nerve root during follow-up**

	**Baseline score**			**Change score during follow-up**			**Follow-up score**		
**Back surgery during follow-up**	ODI*	Leg pain*	Backpain*	ODI*	Leg pain*	Back pain*	ODI**	Leg pain**	Back pain**
Yes	40.4 (15.5)	70.8 (25.8)	64.1 (21.6)	34.2 (13.2)	56.0 (26.4)	49.8 (26.1)	9.7 (10.8)	18.5 (31.9)	17.5 (22.8)
No	28.4 (12.1)	47.6 (23.2)	45.1 (23.8)	11.7 (14.7)	23.5 (29.9)	13.2 (28.4)	16.3 (13.4)	23.6 (24.9)	29.1 (24.2)

Baseline, change and follow-up scores for ODI, VAS leg pain and VAS back pain. Numbers are mean with SD in brackets; P values are for the between group differences.

*P < 0.05.

**Not significant.

## Discussion

The main finding of this study is that lower age, higher education, working full-time and low fear avoidance beliefs each predict a better outcome of chronic unilateral lumbar radiculopathy. Specifically, lower age and low fear avoidance predict a better functional outcome and less back pain, while higher education and working full-time predict less leg pain.

This study also shows that the prognosis for patients referred to multidisciplinary back clinics for chronic unilateral lumbar radiculopathy is good. A total of 75 (64.7%) patients at follow-up had an ODI score below 20, 54 (46.6%) had a VAS leg pain score below 17.5 and 47 (40.5%) had a VAS leg pain score below 22.5.

Identification of prognostic factors predicting persistent pain and disability is important for better understanding of the clinical course – information that can be provided to patients and physicians – and decision-making in treatment and guidance of patients with radiculopathy. We identified higher age and reflex impairment as prognostic factors for non-success, and higher education, working full-time and low fear avoidance as prognostic factors for a successful outcome. These prognostic factors may be used by clinicians to inform patients about the one-year prognosis of chronic unilateral lumbar radiculopathy. In addition, studies show that high fear avoidance can be reduced with cognitive intervention with the prospect of improved outcomes [45-47].

Prognostic research is aimed at using multiple variables to predict the outcome as accurately as possible [33]. Reviews show, however, that most previous studies suffer from methodological weaknesses, which may explain why consistent predictors have not been identified [17,18]. This implies a careful study design and use of multivariable analysis to determine adjusted and independent risk factors for different outcomes, often expressed as probabilities or Odds Ratios. Few studies meet these requests. A single predictor or variable rarely gives an adequate estimate of prognosis.

Two recent studies have explored prognostic factors for outcome of radiculopathy using a multivariable approach. A Norwegian prospective observational multicentre cohort study used the Maine Seattle Back Questionnaire, which is equivalent to the ODI, as the primary outcome measure [7]. The authors used clearly defined cut-off values for non-success validated against the 7-point Likert scale of global perceived recovery. Another randomized controlled study comparing surgery versus prolonged conservative treatment used a similar method [48]. In these studies, the regression analyses were not adjusted for baseline pain scores. Unfortunately, differences in inclusion criteria and categorization of possible predictors complicate comparisons between these two studies and the present study, despite concurrent definitions of successful outcomes. Our study and the study of Lequin et al. [48] both identified lower age as a predictor for success, while other results were conflicting. Accordingly, further methodological standardization is necessary before predictors for the prognosis of sciatica can be validated across studies.

In addition to the main findings in our study, the presence of muscle weakness at baseline predicted a better outcome on the secondary outcome measure VAS leg pain, while the presence of reflex impairment predicted the contrary. The study by Haugen et al. [30] observed the same effect of reflex impairment, while muscle weakness predicted non-success in their study. Again, comparisons are difficult because in the study by Haugen et al., 44.5% of the patients had muscular weakness and 46.2% reduced reflexes at baseline, while the corresponding figures in our study were 81.0% and 47.4%, respectively. Obviously, the patient populations are not directly comparable despite similar inclusion criteria.

Surgically treated patients had more complaints at baseline and improved more during follow-up than those treated non-surgically, but after 52 weeks there were no differences in outcomes between the two groups. Those who had intolerable symptoms seem to benefit from surgery due to rapid pain relief. In previous studies, patients selected for surgery had more disability and pain (higher baseline scores) and more rapid decline of symptoms than those not operated on [49,50]. However, the outcomes at one-year follow-up were similar, which is in agreement with our findings [51,52].

It is a strength that we analysed multiple clinically relevant variables using a multivariable method. Our study is limited by a relatively small number of patients, which precluded explorative analysis of the effect of different combinations of predictors [53,54]. We chose to analyse 15 possible predictors, and thereby exceeded the generally accepted recommendation of a minimum of 10 events per tested predictor [50]. In our multivariable analyses, only 5–8 predictors were included. It is a weakness that this approach entails a risk for type 1 error.

Many previous prognostic studies of chronic radiculopathy have focused on patients encountered in primary care or at the surgical units. The present study deals with patients referred to outpatient multidisciplinary back clinics. Our results should not be generalized to surgical patient populations or to patients from unselected primary care.

## Conclusions

We found that lower age, higher education, working full-time and low fear avoidance beliefs each predict a better outcome of chronic unilateral lumbar radiculopathy. Specifically, lower age and low fear avoidance predict a better functional outcome and less back pain, while higher education and working full-time predict less leg pain. These results should be validated in further studies before being used to inform patients. Unfortunately, comparison with results from two other recent studies conducted with similar methods was difficult because of minor differences in inclusion criteria and categorization of possible predictors. Accordingly, rigorous standardization of the methodology is necessary for future studies before reliable predictors can be identified across studies.
